# Cases Occurring at Union Chapel Hospital 1862

**Published:** 1864-02

**Authors:** W. H. Butler

**Affiliations:** Act. Asst. Surgeon U. S. A.


ART. II.—*Cases occurring at Union Chapel Hospital* 1862. BY W. H. BUTLER, *Act. Asst. Surgeon U. S. A., late in charge.*

*Pernicious Fevers.*—Alexander Craig, aged 26, Co. E, 32d N. Y. Vols. was admitted to Union Chapel Hospital July 7th, 1862. He was then suffering from debility consequent on chronic diarrhœa, which he said had troubled him for several months previously. After being in the house a few days he had a return of the diarrhœa, which was checked to some extent by opium, tannin, acetate of lead, etc., in various combinations. Quinia, brandy, rheubarb and bitters were given as tonics and appetizers. He seemed to be, and was, getting better; went about the house daily, with few exceptions. August 3d, was apparently going on very well, had no fever, but had some looseness of the bowels. For this opium and rheubarb ăă, gr. i, every four hours were given, as these remedies had operated well before, to check the diarrhœa. About midnight he was taken with a bad diarrhœa, and between that hour and seven A. M. of the 4th, had several stools of a watery character. On the way to the ward after the last evacuation he was seized with cramps, pain in the bowels, his lips appeared livid, eyes sunk, cold sweat all over the body, body cold and purplish, breath cold, general collapse, The fingers and hands looked as if they had been soaked in water. Inspirations slow and prolonged, apparently made by a double effort. Breathing 20 per minute. Pulseless; was given at once spts. vini galleii ℥ij, and every 20 minutes ℥i, with friction to all the extremities. Seemed to be dying; said he felt as if he was. Eight and a half o'clock he was given brandy ℥i, capsicum gr. ij, and rubbed with whisky and water, and then enveloped in sheets wrung in hot water, with bottles of hot water to his feet. At nine o'clock he took brandy ℥i, tr capsicum and camphor ăă ʒi. Nine and a half, sulphuric ether ʒss. He had a rice water discharge, and after, was given starch enema with tr. opii ʒi, asafœtida gr. x. Up to this time none of the remedies seemed to have the slightest effect. Ten o'clock, still collapsed, cold and pulseless. ℞ carbonate ammonia gr. v, to be repeated in two hours. After the second dose he seemed to revive some; had a faint pulse, 130 per minute. Has complained some of thirst, and wants water; still talks in a low whisper. The ammonia was repeated at three o'clock P. M., with a little beef tea at intervals, but all nourishment seemed repugnant to him. ℞ at four P. M. S. quinia in sol. gr. iv, every three hours. He seemed better at seven, and was given some beef essence, which he immediately vomited, and soon after had an involuntary movement of the bowels, very watery, and containing patches or flakes of white mucus. An enema of starch with tr. opium and plumbi acetas grs. iij, was thrown up the rectum; this he retained a short time.

Eight and a half P. M.; with a view of controlling the irritability of the stomach, the following was given: ℞ hydrag chl. mite gr. i, opii pulv. gr. ss, every three hours. To have carbonate ammonia gr. v, if he feels faint; beef essence given at 12 M., and 4 o'clock A. M. He was carefully watched, fed at times, as he would seem to rouse, with weak brandy sling, and kept warm, but at seven o'clock of the 4th, a sudden change for the worse took place and he died at eight o'clock, living twenty-five hours from period of attack.

*Gun-shot wound of Shoulder—Removal of Acromion Process.*—William Evritt, aged 22, private 83d Pennsylvania Vols., generally healthy, was wounded at the battle of Malvern Hill. Ball entered in front, two inches from outer aspect of left shoulder, passing apparently below clavicle, between coracoid and acromion processes, shattering the acromion at its junction with spine of scapula. The ball was extracted over spine of scapula six days after, the surgeon having simply to make an incision. No cloth or bone removed. Was admitted to Union Chapel Hospital, Washington, D. C., July 7th, 1862. Wound had simple dressing, little inflammation present, considerable pain on motion.

July 17th. Patient has done pretty well up to date, but for four days past he has complained of pain which required opiates for its relief. Dr. Coolidge, Medical Inspector U. S. A., assisted by Drs. Bliss, U. S. V. Page, U. S. A., Stone, Bigelow. Kennon and Butler, removed the acromion. Patient under ether. An incision was made from one wound to the other, and the broad flap turned over the shoulder, The acromion was dissected out and the spine cut close down to remove all roughness, and tapered well back by bone forceps. Two arteries required ligatures. The flap was brought together with four deep stitches, and supported by broad strips of adhesive plaster going to the opposite shoulder.

July 25th. All going on well; wound is strapped once daily; the cloths changed as often as required, when the wound is gently sponged. Cold water is kept on sufficient to keep it moist as it feels grateful. There has been some tendency of the pus to burrow under the common integument, both before and behind, which is prevented by placing compresses of lint, so the adhesive plaster shall firmly compress the two walls. This tendency was noticed on the twenty-first, and is almost entirely removed to-day, This patient's wound healed slowly, and he was discharged September 3d, 1862, having only moderate use of arm; shoulder quite weak.

*Gun-shot wound Shoulder—Death by Pyemia.*—William Fuller, Co. F, 18th Massachusetts Vols., wounded August 30th, 1862, battle of Bull Run; entered the hospital August 31st. Appearances—circular wound of arm, four inches below acromion process of scapula; shoulder swollen, complains of pain on motion, some yellowish discoloration over outer part of scapula, and tenderness on pressure; applied cold water constantly, which had the effect to reduce the swelling.

Sept. 3d. Cut down on outer edge of scalpula about two inches below acromion process and extracted part of a musket ball greatly misshaped and irregular.

Sept. 5th. Removed another piece of ball from over the scapula, and one inch towards the spine of back in a separate channel; also a few pieces of comminuted bone, apparently of outer edge of scapula; this proceeding lessened the pain and irritation which had been constant, and soon after the swelling was greatly reduced; wound left open and has discharged a very offensive sanious matter since.

Sept. 15th. Several small pieces of bone found in the dressing this morning, brought away by the copious discharge during the night. Has complained of chill and fever. ℞ S. quinia gr. x, opii gr. i, night and morning; best diet, mutton broth, etc.

Sept. 17th. Tongue considerably coated this forenoon. ℞ hydr. mite gr. v, ext. colocynth gr. v. Had a moderate evacuation from bowels and reported better the next day.

Sept. 18th. Some tendency to fever. ℞ S. quinia gr. x, opii puvl. gr. i, night and morning. Has an anxious look.

Sept. 19th. Is better; more appetite; continued quinia and opii; good diet, oyster broth, etc. Wash the wound sol. chl, zinci and water, to be dressed four times daily. Wound open and discharging freely. To have beer ℥viij, three times; is very yellow, and has daily a chill lasting an hour or more in the morning, followed with fever; complains of the noise of the house and of being nervous; is sleepy and dosy, but roused easily; mutters some at night; lays in a heavy typhoid state; talks rather thick; evidently the result of pus absorption.

Sept. 20tn. Quinia gr. x, opii gr. i, at 11 o'clock; best diet, eats pretty well. Opii gr. i, quinia gr. vi, bed time.

Sept. 21st. Tinct. ferri, mu. chl. gutt. xv. 3t. vin. alb. ℥j, 3t., liq. ammo. acetas ʒii 3t., quinia gr. x, opii gr. i, nt. and am.

Sept. 22d. Combined tinct. ferri chl. and spts. minderin, opii gr. i, bed time.

Sept. 23d. Has had hæmorrhage during the night. Vin. alb. ℥i, ovum. vit. i, every four hours, cont. iron and wine through the night.

Sept. 24th. Had some hemorrhage during the night, though controlled yesterday by bandages. ℞ Tinct. ferri, porter, best diet, etc. Died September 25, 1862, of pyæmia. Post mortem 11 o'clock A. M. Found great quantity of serum in pleural cavity and in pericardium. The lower lobe of left lung presented purple, green and red spots—a mottled appearance, and a gangrenous spot about three inches in circumference on lower margin; small points of pus all through the substance of lungs, and a considerable quantity oozed out on pressure. Apex of left lung ulcerated at the point, and was adherent to the ribs—evidences of recent pleuritis on left side. Found old bands of adhesion on upper part of right lung, and evidences of inflammation. Liver—external appearance; color normal, though greatly enlarged, estimated to weigh seven pounds. Cuts firm and looks healthy. The left lobe extended well up against the fifth rib of left side. Gall bladder nearly empty; seemed atrophied. Intestines—stomach healthy; spleen seemed somewhat enlarged and somewhat softened. Kidneys normal. Heart, left side empty; right auricle filled with a fibrinous clot running up into veins of head. Found fracture of scapula caused by the ball, extending from right to left, just below spine and coracoid process—two parts completely severed, and in the centre considerably comminuted. Blood had been effused under nearly the whole extent of the scapula, running back as far as spine of back. the muscles disorganized and greenish. Found a great pocket of coagulated blood running down left arm and all about head of humerus. The muscles for considerable extent in this locality were disorganized and black.

